# CRISPR-CBEI: a Designing and Analyzing Tool Kit for Cytosine Base Editor-Mediated Gene Inactivation

**DOI:** 10.1128/mSystems.00350-20

**Published:** 2020-09-22

**Authors:** Haopeng Yu, Zhaowei Wu, Xiangdan Chen, Quanjiang Ji, Shiheng Tao

**Affiliations:** a College of Life Sciences and State Key Laboratory of Crop Stress Biology in Arid Areas, Northwest A&F University, Yangling, Shaanxi, China; b Bioinformatics Center, Northwest A&F University, Yangling, Shaanxi, China; c School of Physical Science and Technology, ShanghaiTech University, Shanghai, China; Northern Arizona University

**Keywords:** base editing, cytosine base editor, CRISPR, gene inactivation

## Abstract

Base editing is a promising technique, allowing precise single-base mutagenesis in genomes without double-strand DNA breaks or donor templates. Cytosine base editors (CBEs) convert cytosine to thymidine. In particular, CBEs can transform four codons, CAA, CAG, CGA, and TGG, into stop codons, providing a new means to rapidly inactivate a gene of interest and enabling loss-of-function study in recombination-deficient species and the construction of gene-inactivation libraries. However, designing single guide RNAs (sgRNAs) for gene inactivation is more complicated and more restricted in applicability than using the lustered regularly interspaced short palindromic repeats (CRISPR)/CRISPR-associated (CRISPR/Cas9) system only, especially for researchers who do not specialize in the bioinformatics skills needed to design and evaluate sgRNAs. Here, we present a new user-friendly designing tool kit, namely, CRISPR-CBEI (cytosine base editor-mediated gene inactivation), including a Web tool and a command-line tool. The Web tool is dedicated to the design of sgRNAs for CBE-mediated gene inactivation and integrates various functions, including open reading frame (ORF) identification, CBE customization, sgRNA designing, summarizing, and front-end off-target searching against user-defined unlimited-file-size local genome files without the necessity of uploading to the server. The command-line version serves the same purpose but for a larger number of coding DNA sequences (CDSs), for instance, for designing a CBE-inactivation library in a target species which provides comprehensive evaluations of CBEs and target genomes. We envision that this tool would contribute to CBE-inactivation design.

**IMPORTANCE** Life science has been in pursuit of precise and efficient genome editing in living cells since the very beginning of the first restriction cloning attempt. The introduction of RNA-guided CRISPR-associated (Cas) nucleases contributed to this ultimate goal through their ability to deliver a double-strand break (DSB) to a precise target location in various species, obsoleting the preceding editing tools, such as zinc-finger nucleases (ZFNs) and transcription activator-like effector nucleases (TALENs). The derivative technology, base editing, combines the catalytically inactivated Cas nuclease and nucleotide deaminase and mediates the genetic modifications at single-nucleotide precision without introducing a DSB. Moreover, the cytosine base editors (CBEs) are able to transform multiple codons into stop codons, rapidly inactivating a gene of interest and enabling loss-of-function study in some recombination-deficient species. Here, we present the CRISPR-CBEI tool kit to assist the design of sgRNAs for CBE-mediated gene inactivation.

## Abstract (precis)

Life science has been in pursuit of precise and efficient genome editing in living cells since the very beginning of the first restriction cloning attempt. The introduction of RNA-guided CRISPR-associated (Cas) nucleases contributed to this ultimate goal through their ability to deliver a double-strand break (DSB) to a precise target location in various species, obsoleting the preceding editing tools, such as zinc-finger nucleases (ZFNs) and transcription activator-like effector nucleases (TALENs). The derivative technology, base editing, combines the catalytically inactivated Cas nuclease and nucleotide deaminase and mediates the genetic modifications at single-nucleotide precision without introducing a DSB. Moreover, the cytosine base editors (CBEs) are able to transform multiple codons into stop codons, rapidly inactivating a gene of interest and enabling loss-of-function study in some recombination-deficient species. Here, we present the CRISPR-CBEI tool kit to assist the design of sgRNAs for CBE-mediated gene inactivation.

## INTRODUCTION

Clustered regularly interspaced short palindromic repeats (CRISPR) and CRISPR-associated (Cas) systems are regarded as adaptive countermeasures in prokaryotes against foreign DNA invasion (1, 2). The RNA-guided Cas nucleases have been developed as versatile and multipurpose tools for genome editing in a number of species (3–9), through their ability to cleave double-strand DNA precisely and in a programmable manner (1). In most eukaryotes and a few prokaryotes, a double-strand break (DSB) can be rapidly repaired by nonhomologous end-joining (NHEJ) machineries in an error-prone manner with the introduction of insertions, deletions, translocations, or other DNA rearrangements, eventually resulting in gene disruption (10, 11). More-precise gene editing could be achieved by taking advantage of the homology-directed repair (HDR) mechanism in host cells via supplementing a donor template (12, 13).

**FIG 1:**
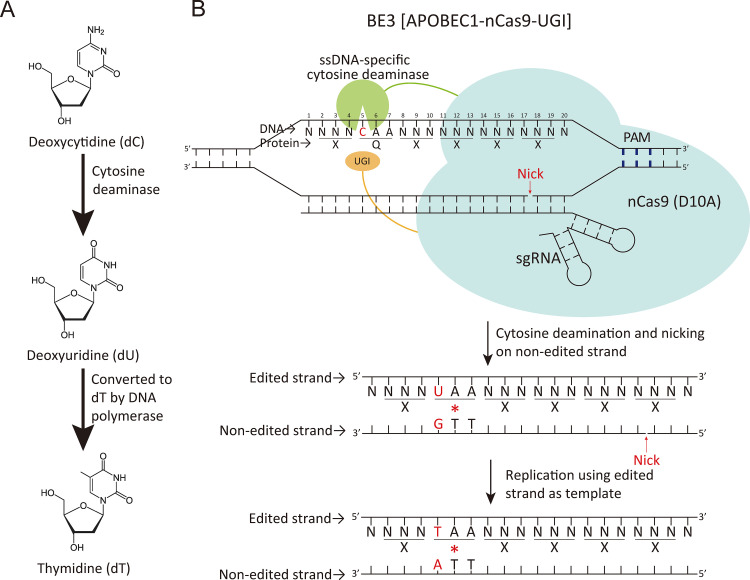
(A) Cytosine deaminase first converts the deoxycytidine (dC) to deoxyuridine (dU), and then the replication machinery in cell replaces the deoxyuridine (dU) with thymidine (dT). (B) BE3 (APOBEC1-nCas9-UGI) was used as an example to explain the concept of cytosine base editor-mediated gene inactivation. A single-stranded DNA (ssDNA)-specific cytosine deaminase (APOBEC1) and a uracil glycosylase inhibitor (UGI) were fused to the N terminus and C terminus of nCas9 (containing a D10A mutation, working as a nickase), respectively. An sgRNA guides the nCas9 to specify a target location in a specific gene, which then nicks the nonedited strand. The cytosine deaminase catalyzes the deamination reaction of dC in an editable codon, CAA, transforming the dC to dU. The UGI protected the edited site from the intrinsic mismatch repair machinery. The DNA polymerase replaces the nucleotide from the nick created by nCas9, and the dU is further substituted to dT, thus generating a stop codon, TAA.

The recent development of base editors has enabled gene editing at single-nucleotide (nt) precision without creating any double-strand break during the process (14, 15). To date, two major types of base editors have been developed by combining nucleotide deaminases and catalytically inactivated Cas nucleases or Cas nickases (16). The adenine base editors (ABEs) transform deoxyadenosine (dA) to deoxyguanosine (dG) (17). The cytosine base editors (CBEs) convert deoxycytidine (dC) to thymidine (dT) (Fig. 1A) (14). Base editors enable treatment of genetic diseases caused by single-nucleotide polymorphism (16). In particular, CBEs potentially convert four types of codons, CAA, CGA, CAG (on the sense strand of a coding DNA sequence [CDS]), and TGG (on the antisense strand of a CDS), into stop codons (Fig. 1B). This feature is further applied to inactivate target genes in host cells, opening an avenue for rapidly constructing loss-of-function mutations and gene-inactivation libraries in recombination-deficient or NHEJ-deficient species, such as Acinetobacter baumannii, Klebsiella pneumoniae, Pseudomonas aeruginosa, and Staphylococcus aureus (7, 8, 18, 19). However, the design of single guide RNAs (sgRNAs) for CBE-mediated gene inactivation is more complicated and narrower in its range of applications than that employed for Cas nuclease-derived DNA cleavage. Generally, the designing workflow consists of four steps: (i) pinpointing the location of the target coding sequence within the input DNA sequence; (ii) identifying all possible flanking spacer sequences with legitimate protospacer-adjacent motifs (PAMs) within the coding sequence; (iii) selecting the spacers with at least one editable deoxycytidine in the editing window; and (iv) checking whether the editable dC belongs to the four types of codons, i.e., CAA, CGA, CAG, and TGG. Moreover, some additional criteria should also be considered, including the surrounding sequence preferences of CBEs, the location of the spacer, and, notably, the potential off-target effect of the selected spacer. However, many researchers in the biochemical field or biomedical field do not have the bioinformatics expertise to search for and analyze sgRNAs for CBE-mediated gene inactivation efficiently. Furthermore, although there are a few software programs designed for searching sgRNAs for CBEs or ABEs, no current software specializes in identifying sgRNAs for CBE-mediated gene inactivation (20).

Here, we present a new computational tool kit, namely, CRISPR-CBEI, facilitating the design of sgRNAs for cytosine base editor-mediated gene inactivation. This tool kit contains a user-friendly Web-based tool for searching and analyzing sgRNAs on single or multiple coding sequences. The searching parameters, including spacer length, PAM sequence and location, editing window size, and searching region, are flexibly tunable. The off-target verification that follows enables the front-end search against user-defined genome files without the necessity of uploading to the server. Through optimizing the algorithm, the asynchronous front-end off-target search becomes fast and requires less memory, with no limit on the input file size. In parallel, a local Python-based version is also provided that is designed to achieve the same goal but is specifically configured for processing large numbers of coding sequences. The resulting sgRNA library can contribute to the construction of a CBE-mediated gene-inactivation library. Moreover, this tool is further explored for the evaluation of the inactivation ability of CBEs and the inactivation potential of a genome.

## RESULTS

### CBEI designing on the Web.

**FIG 2:**
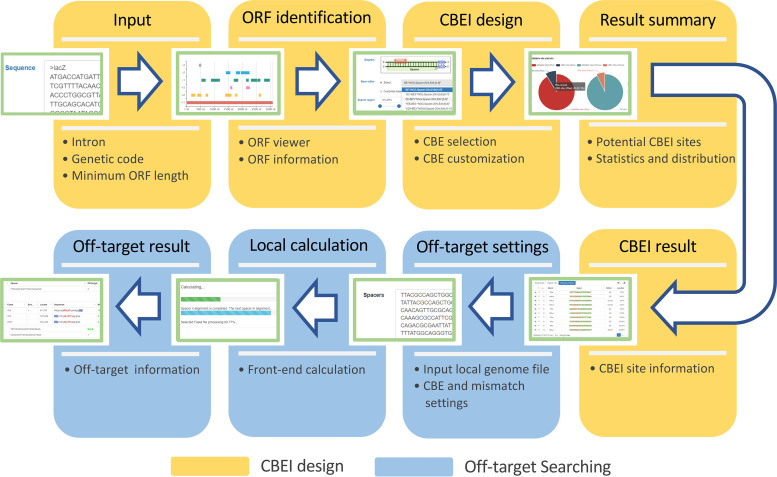
Workflow of the Web-based CRISPR-CBEI tool kit.

To facilitate the designing of sgRNAs for cytosine base editor-mediated gene inactivation, we created a front-end user-friendly designing tool, namely, CRISPR-CBEI, by using JS script, HTML5, and CSS, guiding by simplified operations and direct navigation, supporting fully customizable parameters in all processes, and providing results in lists and statistics in interactive graphics (Fig. 2). To design sgRNAs for CBE-mediated gene inactivation, the Web-based CRISPR-CBEI tool requires the user to submit the target CDS via the “Input sequence” box. Input of single sequences either in plain text or in the standard fasta format is acceptable, whereas, for multisequence input, it is mandatory to use the fasta format to separate the sequences. Next, the input sequence is subjected to an open reading frame (ORF) detection procedure to identify the location of the target coding sequence. If the input sequence contains one or more introns, then the user should specify the base spans of all exons. In addition, the ORF detection module fully supports customization for 25 types of genetic codes and all initiation and stop codons. The minimal ORF detection length is set to 75 bp by default, with a customizable range of 24 bp to 150 bp. The results of ORF detection are represented in the form of interactive graphics, showing all identified ORFs on 6 frames (−3, −2, −1, +1, +2, and +3) by rectangles with different colors. The details of the information corresponding to each ORF automatically appear when the mouse is pointed to the corresponding rectangle. In addition, all ORF information is listed in a table below the graphic. Either clicking a rectangle on the graphic or selecting an ORF in the table specifies a target coding sequence for the subsequent CBEI design process. Note that when multiple sequences are submitted, the ORF detections proceed for each one individually. Users can select the sequences on the drop-down list for the next procedure one by one. Then, the user should specify the type of cytosine base editor. The Web tool integrates 13 types of commonly used CBEs in a drop-down list. In addition, we also support customization by the user of the parameters of CBEs, including changing the PAM sequences (degenerate bases are also supported by using the regular expression parameters), the length and location of the spacer, and the editing window size and location. The alterations in CBE parameters are schematically visualized within the CBE diagram in real time. By default, spacers are searched within the top 50% of the body of the coding sequence to ensure complete inactivation of a gene, although the user can adjust the search region parameters freely. After that, the spacer search begins upon clicking the “CBEI design” button. The Web tool first indicates the CBEI-available site numbers in the whole coding sequence and in the defined search region and gives informational details of the site with the most potential (the first available site in the coding sequence) in a table. Among the spacer details, green nucleotides within braces indicate the spacer, wheat-colored nucleotides within brackets specify the editing window, and red nucleotides in parentheses show the editable site and the changing pattern. Outside the spacer, blue nucleotides indicate the PAM sequence. Codons are separated by commas. To enable viewing of more information corresponding to potential CBEI spacers, the Web tool provides graphical statistics, including a pie chart to indicate the fraction of CBEI sites in all spacers on the plus and minus strands of the coding sequence and a histogram to show the distribution of the CBEI sites on the coding sequence. Detailed information from all CBEI spacers is listed in the table below, supporting multiformat export. Notably, the local sequence context adjacent to the CBEI site usually affects the activity of CBE, generally following the order TC ≥ CC ≥ AC > GC (16, 18). This information is also included in the spacer list.

### Off-target searching.

The off-target effect is a major concern in using CRISPR-based gene-editing technology. Generally, Cas nucleases require a perfect match between the target sequence and the spacer within the seed region (usually the eight nucleotides flanking the upstream portion of a PAM for SpCas9 [Streptococcus pyogenes Cas9]) to trigger its DNA binding activity. However, a few mismatches outside the seed region within the spacer are often permissible but their presence is associated with a significantly reduced chance of arming the nuclease activity of Cas nucleases (21–23). Consequently, the resulting sgRNAs should be verified by an off-target search against the genome corresponding to the target sequence. In this study, a front-end off-target searching module was created by adopting a divide-and-conquer algorithm to boost computational efficiency and reduce the amount of memory occupied (about 200 Mb) (see Fig. S1 in the supplemental material). sgRNAs from the previous CBEI design step are used as input. When the base editor information, genome file, and mismatch number are specified, the off-target search starts upon clicking the “Predict” button. Notably, this tool has no limit in the genome file size, as any file larger than 50 Mb is automatically sliced into 50-Mb segments, which are then subjected to the off-target searching individually (the junction between every two segments is also included in consideration). The whole operation is straightforward and easy to use and does not collect any user information data. The results of off-target searching are presented in a table, supporting multiformat export.

10.1128/mSystems.00350-20.1 FIG S1 Details of the workflow of the front-end off-target searching. Download FIG S1, TIF file, 1.2 MB. Copyright © 2020 Yu et al. 2020 Yu et al. This content is distributed under the terms of the Creative Commons Attribution 4.0 International license.

As a consequence of optimization of the front-end computation algorithm, the time cost of the off-target searching is low. We have performed a benchmark analysis of the front-end off-target searching for six commonly used genomes and their coding sequences (Table 1). The computational efficiency is predominantly affected by the mismatch number and the genome size. By default (using a CPU as i5-3470 at 3.20 GHz), when the mismatch value is set to a value less than or equal to 3, simultaneously searching 10 spacers in 100 Mb of genome data takes about 11.91 s (or 1.19 s per spacer per 100 Mb). Therefore, the off-target prediction module of CRISPR-CBEI took only seconds to complete the off-target search for 10 spacers among the species with a smaller genome, such as Escherichia coli, Saccharomyces cerevisiae, *and* Caenorhabditis elegans, no matter how many mismatches were set (Table 1). Working with the species with larger genome size, for instance, the human genome, searching the off-target sites for 10 spacers with 0 mismatches required 108 s (10.8 s per spacer per human genome), while the time cost increased to 5 min 52 s when the mismatch value reached a total of 3 (35.2 s per spacer per human genome).

**TABLE 1:** Benchmark of the front-end off-target searching (simultaneously searching 10 spacers against the target genome on a platform using i5-3470 at 3.20 GHz)

Species	Genome size (Mb)	Processing time required for indicated number of mismatches^*a*^			
		≤3	≤2	≤1	=0
Escherichia coli CDS	4.66	0m1s ± 0.01s	0m1s ± 0.02s	0m0s ± 0.01s	0m0s ± 0.02s
Escherichia coli DNA	4.50	0m1s ± 0.01s	0m0s ± 0.02s	0m0s ± 0.02s	0m0s ± 0.01s
Saccharomyces cerevisiae CDS	11.10	0m1s ± 0.04s	0m1s ± 0.03s	0m1s ± 0.03s	0m0s ± 0.01s
Saccharomyces cerevisiae DNA	11.79	0m1s ± 0.04s	0m1s ± 0.03s	0m1s ± 0.03s	0m0s ± 0.01s
Caenorhabditis elegans CDS	52.00	0m6s ± 0.20s	0m4s ± 0.13s	0m3s ± 0.19s	0m2s ± 0.01s
Caenorhabditis elegans DNA	97.24	0m12s ± 0.24s	0m8s ± 0.24s	0m6s ± 0.26s	0m4s ± 0.02s
Danio rerio CDS	90.86	0m11s ± 0.23s	0m7s ± 0.04s	0m5s ± 0.21s	0m3s ± 0.01s
Danio rerio DNA	1,304.19	2m35s ± 3.57s	1m46s ± 3.12s	1m13s ± 0.28s	0m47s ± 0.25s
Mus musculus CDS	98.64	0m12s ± 0.41s	0m8s ± 0.25s	0m6s ± 0.25s	0m3s ± 0.01s
Mus musculus DNA	2,642.60	5m7s ± 7.03s	3m33s ± 6.42s	2m32s ± 6.24s	1m35s ± 0.05s
Homo sapiens CDS	146.49	0m18s ± 0.01s	0m12s ± 0.30s	0m8s ± 0.36s	0m5s ± 0.03s
Homo sapiens DNA	2,994.31	5m52s ± 7.89s	4m2s ± 7.02s	2m52s ± 7.04s	1m48s ± 0.20s

a m, minutes; s, seconds.

### Extensive CBEI designing and evaluation.

**FIG 3:**
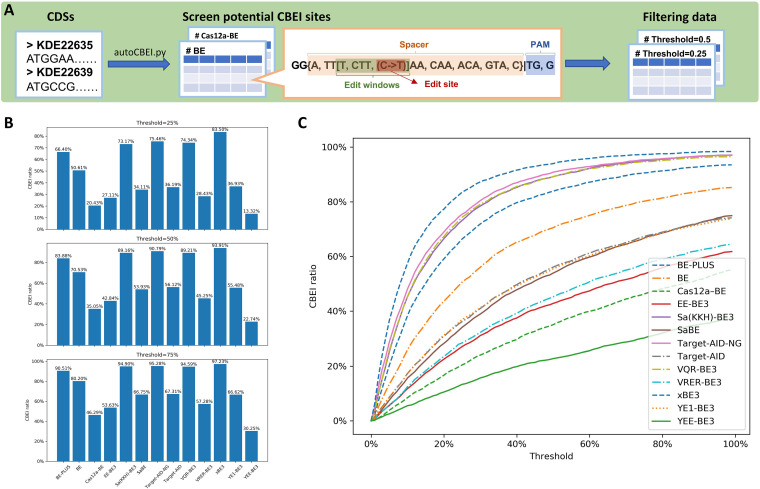
Overview of the Python-based command-line tool and evaluations of the CBEI abilities of different CBEs. (A) The working pipeline of the “autocbei.” (B) The CBEI ratios of 13 types of CBEs within different search regions (top 25%, 50%, and 75% of the CDS body) using the whole-CDS data of Bacillus subtilis. (C) Analyses of the CBEI abilities of 13 types of CBEs using the same data set.

In parallel, a command-line version, namely, “autocbei,” was also created based on Python scripts, allowing the user to process large amounts of data, such as a complete set of coding sequences in a species. The extensive CBEI design procedure starts by using only one command (Fig. 3A). By default, the Python script includes all 13 types of commonly used CBEs and supports customization of CBEs. This tool calculates the potential CBEI editing sites on each coding sequence for each of the selected CBEs, outputting three sets of results according to different search regions corresponding to the top 25%, 50%, and 75% of the body of the CDS, respectively. Each of the sets of results contains a text file that includes information of all CBEI spacers and a set of statistical graphics, comprising the analyses of all input coding sequences, and the evaluations of each CBE. Therefore, this tool also enables the user to assess the CBEI ability of each CBE and the CBEI potential of the target species.

In this study, we defined the CBEI ability as the ratio of CBEI-available CDSs to all CDSs in a species corresponding to a fixed search region. As shown in Fig. 3B, The CBEI abilities differed greatly in various CBEs, owing to their specific editing criteria. Target-AID-NG and xBE3 had the best CBEI abilities to potentially disrupt over 90% of coding sequences in the genome of Bacillus subtilis within a search region consisting of the top 50% of the CDS body since their PAM requirements are the most minimal (needing only the NG dinucleotide) (Fig. 3B). In contrast, in the same 50% search region, YEE-BE3 was able to inactivate only 23% of CDSs in B. subtilis, whose editing window is extremely narrow (only position 6 of the spacer is editable) (Fig. 3B). BE-PLUS, Sa(KKH)-BE3, and VQR-BE3 shared similar levels of CBEI abilities ranging from 84% to 89% (Fig. 3B), while other CBEs, such as Cas12a-BE, EE-BE3, SaBE, Target-AID, VRER-BE3, and YE1-BE3, showed intermediate levels of CBEI abilities ranging from 35% to 71% (Fig. 3B). For better visualizations and comparisons of the CBEI abilities among the CBEs, we have integrated an automatic analysis program in “autocbei.” As shown in Fig. 3C, the closer the slope to the left upper corner of the figure, the better the CBEI ability of the CBE. For example, xBE3 was able to inactivate the greatest number of CDSs in the smallest search region. However, even with the search region expanded to 100% of the CDS body, the CBEI ability of YEE-BE3 never reached 40%.

**FIG 4:**
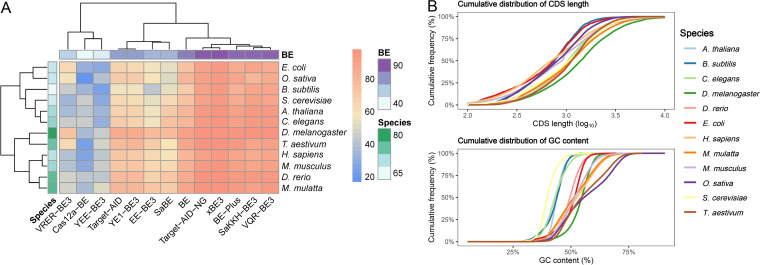
(A) Analyses of the CBEI abilities of different CBEs and the CBEI potentials in 12 model species under a fixed CBEI search region at the top 50% of the CDS body. (B) Cumulative distribution analyses of the CDS length and GC content in 12 model species.

In addition, the genetic contents of genomes in various species also affect the CBEI ability of each CBE. As shown in Fig. 4A, we systematically compared the CBEI abilities in 12 model organisms within a fixed search region set as the top 50% of the CDS body. Target-AID-NG, xBE3, BE-PLUS, SaKKH-BE3, and VQR-BE3 showed less effect from the target genome specificity. However, the performance of VERE-BE3 differed highly for various species, working better in the species with relatively higher levels of CDS GC content, such as Drosophila melanogaster, Triticum aestivum, and Oryza sativa (Fig. 4B), since YERE-BE3 adopts NGCG as its PAM recognition sequence. In contrast, Cas12a-BE performed better in species with lower levels of CDS GC content, such as S. cerevisiae, Arabidopsis thaliana, and C. elegans (Fig. 4B), owing to its T-rich PAM specification (TTTV). Furthermore, the “autocbei” tool also allows the user to reveal the CBEI potential of the target species. D. melanogaster was the most competent species with respect to CBEI, with the most minimal restriction in CBE types (Fig. 4A). Moreover, animals usually have higher CBEI potential than bacteria, fungi, metazoans, and plants, since they usually have longer CDSs (Fig. 4B), allowing CBE to identify greater numbers of potential loci for generating pre-stop codons.

## DISCUSSION

In this study, we developed a tool kit, namely, CRISPR-CBEI, that enables users to search spacers of sgRNAs for cytosine base editor-mediated gene inactivation potential. The Web version is capable of processing single or multiple inputs and verifying the results by a front-end off-target searching module. Moreover, the Python-based command-line version handles large amounts of input data, allowing users to analyze the editing potential of CBEs and target genomes. To our knowledge, there are a few tools available for assisting the design of spacers for base editors. However, CRISPR-CBEI is the first tool designed to specify the spacers, which represent the potential to disrupt a target gene by CBE, facilitating CBE-based loss-of-function study and rapid construction of a gene-inactivation library.

Commonly, front-end searching is undervalued as a consequence of lacking support for multithreading computation and graphics processing unit (GPU) acceleration on Web browsers. Therefore, most current off-target searching Web tools provide server-side command-line-based computation against the genome data of a few built-in commonly used species (20, 24, 25). However, such tools lack the flexibility to support off-target searching against user-defined or other uncommon genomes. To reinforce this drawback, some of them also offer local command-line versions, which are usually not user-friendly (26). In this study, we introduced a very efficient front-end off-target searching module within the CRISPR-CBEI Web tool. The searching algorithm has been extensively optimized to ensure its efficiency. The process of searching against most genomes usually finishes in seconds. Other tools usually require more time for uploading inputs, processing data, and downloading results and to accommodate network latency. Even for large genomes such as that of H. sapiens, this front-end off-target searching module needs only about 35 s to process one spacer, which is relatively fast. Remarkably, this benchmark represents processing on a quite obsolete platform with a third-generation core i5 CPU. The time cost could be further shrunk by using a more advanced CPU with higher single-thread performance. According to Moore's law and the continuous development of integrated circuits, the computing power of front-end equipment will gradually improve, which would support the off-target prediction move from the back end to the front end. Thus, we envision that the front-end off-target searching will become predominant.

## MATERIALS AND METHODS

### The CRISPR-CBEI Web tool.

The Web-based CRISPR-CBEI tool is freely accessible at https://taolab.nwafu.edu.cn/crisprcbei/ or https://atlasbioinfo.github.io/CRISPR-CBEI/. It adopts HTML5 as the framework, CSS3 for the layout, JavaScript for the calculation process, and the Web Workers of HTML5 for loading local files. The built-in genetic codes and values in the ORF identification function are based on ORFfinder (https://www.ncbi.nlm.nih.gov/orffinder/). JavaScript (JS) is used to perform the translation of the user-customized base editor parameter into a regular expression. Then, a regular expression-matching algorithm is initiated to search for the potential spacers which perfectly match the regular expression within the user-selected ORF. Subsequently, the program judges whether a deoxycytidine of the four editable codons, CAA, CAG, CGA, and TGG, is present in the defined editing window and whether the editing resulted in a premature stop codon. Finally, the program presents all potential CBEI spacers within the user-defined search region of the target ORF.

### Front-end off-target searching.

HTML Web Workers were adopted in CRISPR-CBEI to enable local data processing without the necessity of uploading large genomic files to the server. The front-end off-target searching first splices the spacer into “mismatch + 1” fragments, termed anchors. A sequence alignment is then started to match the genome with these anchors. If the anchor matches, the program then determines whether its upstream sequence or downstream sequence contains a PAM of the selected CBE. After the PAM determination, the mismatch value representing the remaining part on the spacer is compared with the set value. Spacers that have a lower mismatch value than the pre-set value are considered potential off-target sites. The efficiency of the search process depends on CPU performance, genome size, and the mismatch value. The front-end off-target searching algorithm supports the use of large genome files by breaking the genome file into 50-Mb segments. Accordingly, 100-nt sequences of the upstream and downstream regions flanking the break site are extracted as new segments for the off-target searching process to avoid neglecting the off-target site near the break site.

### The “autocbei” Python-based command-line tool.

The source code and detailed instructions are deposited in Github for free access (see below). This command-line tool is written in Python 3 and requires “biopython” and “matplotlib” packages for running. The “autocbei” command-line tool has been uploaded to PyPI and Anaconda Cloud for easy installation with “pip” and “conda.” More installation and usage information is available on the corresponding Github (https://github.com/atlasbioinfo/CRISPR-CBEI/blob/master/autocbei/README.md), PyPI (https://pypi.org/project/autocbei/), and Anaconda (https://anaconda.org/atlasbioinfo/autocbei) Web pages. We also set up “TravisCI” for continuous integration testing for “autocbei.” CDS data for the 12 model species used in the analyses were obtained from the Ensembl database.

### Support and tutorials.

The CRISPR-CBEI tool kit includes two applications, the Web tool and the command-line tool (“autocbei”), both of which contain detailed instructions. An introduction to the Web version of CRISPR-CBEI can be found on the “Help” page of the website, where we provided a downloadable complete user manual in a PDF file. Detailed information regarding the installation and usage of the “autocbei” tool is provided in the Github “README” file and can also be obtained through the “-h” parameter. If users have problems using both versions of the CRISPR-CBEI tool kit, they can submit a Github issue or contact the project administrator by email. Contact details can be found on the CRISPR-CBE webpage or Github project homepage.

### Data availability.

We provide the source code for the Web-based CRISPR-CBEI tool on Github (https://github.com/atlasbioinfo/CRISPR-CBEI/). The source code and detailed instructions are deposited in Github for free access (https://github.com/atlasbioinfo/CRISPR-CBEI/tree/master/autocbei).
