# Supplementary figures and images for: CRISPR-CBEI: a Designing and Analyzing Tool Kit for Cytosine Base Editor-Mediated Gene Inactivation
Source: mSystems. 2020 Sep 22;5(5):e00350-20. doi: 10.1128/mSystems.00350-20 (PMC7511213; doi:10.1128/mSystems.00350-20)

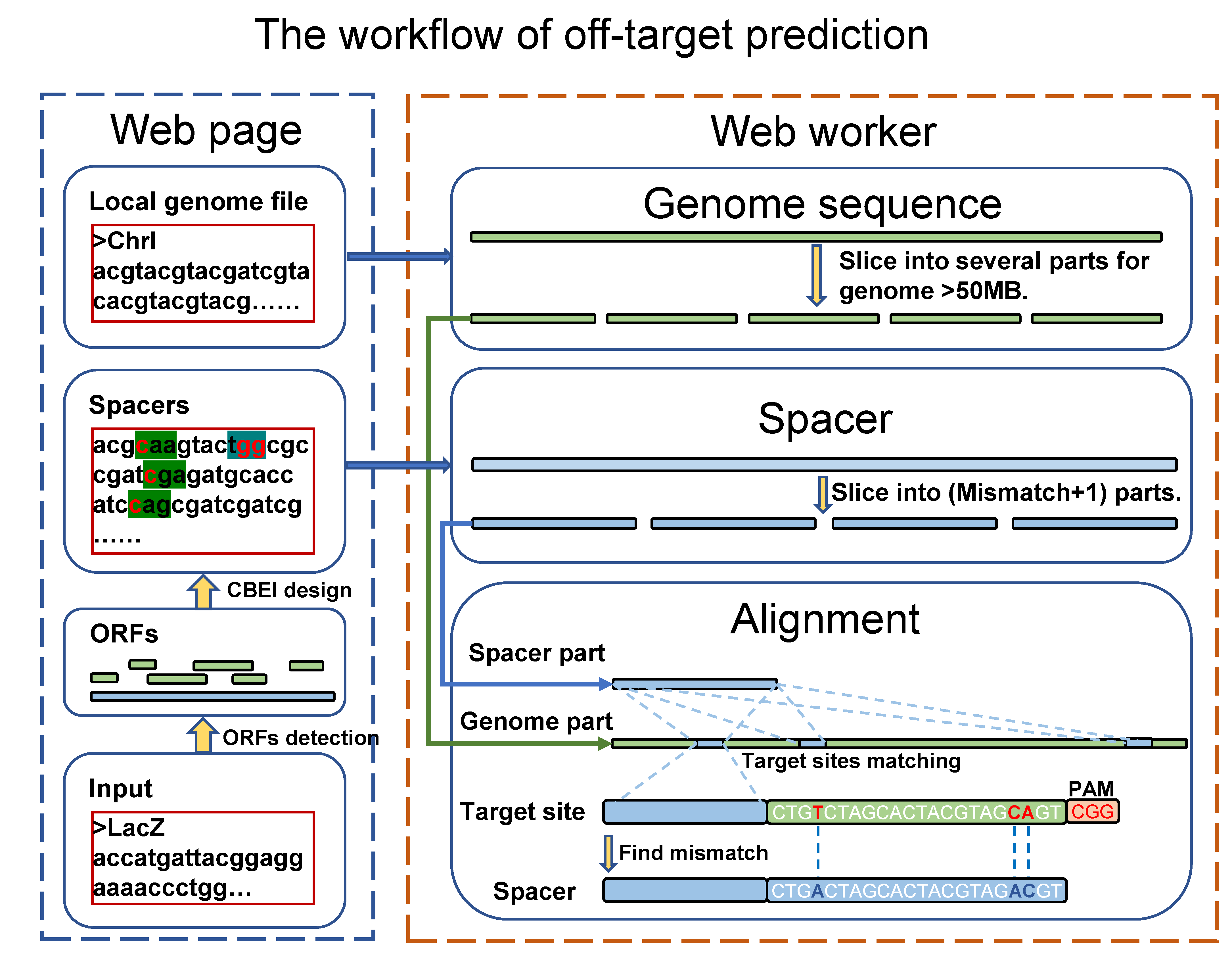

Supplement: FIG S1 [file mSystems.00350-20-sf001.tif]
